# Molecular Cloning and Functional Characterization of Two *Brachypodium distachyon UBC13* Genes Whose Products Promote K63-Linked Polyubiquitination

**DOI:** 10.3389/fpls.2015.01222

**Published:** 2016-01-07

**Authors:** Huiping Guo, Rui Wen, Zhi Liu, Raju Datla, Wei Xiao

**Affiliations:** ^1^College of Life Sciences, Capital Normal UniversityBeijing, China; ^2^National Research Council CanadaSaskatoon, SK, Canada; ^3^Department of Microbiology and Immunology, University of SaskatchewanSaskatoon, SK, Canada

**Keywords:** *Brachypodium distachyon*, Ubc13, K63-linked polyubiquitination, DNA-damage tolerance, stress response

## Abstract

Living organisms are constantly subject to DNA damage from environmental sources. Due to the sessile nature of plants, UV irradiation is a major genotoxic agent and imposes a significant threat on plant survival, genome stability and crop yield. In addition, other environmental chemicals can also influence the stability of the plant genome. Eukaryotic organisms have evolved a mechanism to cope with replication-blocking lesions and stabilize the genome. This mechanism is known as error-free DNA damage tolerance, and is mediated by K63-linked PCNA polyubiquitination. Genes related to K63-linked polyubiquitination have been isolated recently from model plants like *Arabidopsis* and rice, but we are unaware of such reports on the crop model *Brachypodium distachyon*. Here, we report the identification and functional characterization of two *B. distachyon UBC13* genes. Both Ubc13s form heterodimers with Uevs from other species, which are capable of catalyzing K63 polyubiquitination *in vitro*. Both genes can functionally rescue the yeast *ubc13* null mutant from killing by DNA-damaging agents. These results suggest that Ubc13-Uev-promoted K63-linked polyubiquitination is highly conserved in eukaryotes including *B. distachyon.* Consistent with recent findings that K63-linked polyubiquitination is involved in several developmental and stress-responsive pathways, the expression of *BdUbc13*s appears to be constitutive and is regulated by abnormal temperatures.

## Introduction

Plants, due to their sessile nature, have developed unique and efficient mechanisms to cope with many environmental stresses including DNA damage. While sunlight is essential for photosynthesis, the resultant UV irradiation exerts strong influences on plant growth and development, and reduces crop yield by inducing DNA damage. High doses of UV irradiation exerts strong influences on plant growth and development, and reduces the crop yield by inducing DNA damage (Frohnmeyer and Staiger, 2003). To prevent damage from UV irradiation and other genotoxic stresses, plants have evolved a series of molecular mechanisms, including the accumulation of UV-absorbing pigments (flavonoids and hydroxycinnamic acid derivatives; Li et al., 1993; Christie and Jenkins, 1996; Bieza and Lois, 2001; Fabon et al., 2010), the production of reactive oxygen species (ROS) and activation of DNA repair systems (Gallego et al., 2000; Liu et al., 2000, 2003; Sakamoto et al., 2003; Garcia-Ortiz et al., 2004; Takahashi et al., 2005; Liang et al., 2006; Vonarx et al., 2006; Wang and Liu, 2006; Wen et al., 2006; Curtis and Hays, 2007).

In addition to the DNA repair pathways, eukaryotic organisms also cope with replication-blocking lesions by a mechanism traditionally known as DNA post-replication repair (PRR). In *Saccharomyces cerevisiae*, the PRR pathway is initiated by the stable E2–E3 complex Rad6–Rad18. This pathway promotes replicative bypass of lesions encountered instead of removing them. It has been renamed DNA-damage tolerance (DDT) to reflect the fact that it does not actually repair the damage but allows cell survival in the presence of the lesion (Andersen et al., 2008). The DDT pathway includes two branches, error-prone translesion synthesis (TLS) and error-free lesion bypass (Broomfield et al., 2001; Zhang et al., 2011) and they are achieved via sequential ubiquitination of proliferating cell nuclear antigen (PCNA). While PCNA monoubiquitination by Rad6–Rad18 promotes TLS, another E2–E3 complex, Mms2-Ubc13-Rad5, is thought to further ubiquitinate PCNA at the same K164 residue to form a K63-linked polyUb chain that is required for error-free lesion bypass (Hoege et al., 2002; Xu et al., 2015).

Ubc13 is highly conserved in evolution and is the only known Ub-conjugating enzyme dedicated to catalyzing the K63-linked polyubiquitination reaction in eukaryotes. However, such a reaction absolutely requires a Ubc-like, or Ubc/E2 variant (Uev), which forms a stable heterodimer with Ubc13 (Hofmann and Pickart, 1999) and orients an acceptor Ub so that its K63 residue is exposed to the C-terminus of donor Ub at the Ubc13 active site (McKenna et al., 2001). Different from K48- and K11-linked polyubiquitin chains that target proteins for degradation via the 26S proteasome (Kulathu and Komander, 2012), K63-linked Ub chains are thought to be involved in the regulation of a number of cellular signaling pathways. Yeast and mammal genomes contain only one *UBC13* gene, while *Arabidopsis* and Zebrafish genomes contain two *UBC13* genes (Wen et al., 2006; Li et al., 2010). Budding yeast *MMS2* encodes a Uev protein (Broomfield et al., 1998) and, together with Ubc13, is involved in error-free DDT (Hofmann and Pickart, 1999; Brusky et al., 2000). Higher eukaryotic genomes appear to contain multiple *UEV* genes (Xiao et al., 1998; Wen et al., 2008) that may confer different biological functions. For example, in addition to the proposed DDT function, mammalian Uevs may also be involved in innate immunity (Deng et al., 2000), in breast cancer metastasis (Wu et al., 2014) and the specificity relies on different Uev genes (Andersen et al., 2005). In addition to the possible DDT function, *Arabidopsis* Ubc13s are also required for apical dominance (Yin et al., 2007), iron metabolism (Li and Schmidt, 2010), immunity (Mural et al., 2013), and auxin signaling (Wen et al., 2014). To date the roles of Ubc13-Uev mediated K63-linked polyubiquitination in crop models have not been explored despite a recent report of a rice *UBC13* gene (Zang et al., 2012).

*Brachypodium distachyon*, owing to a relatively small genome (300 Mbp), short generation time (8–12 weeks), efficient *Agrobacterium*-mediated transformation and availability of mutant collections, has been regarded as a model species for monocot, temperate cereals, and biofuel plants for over a decade (Draper et al., 2001; Catalan et al., 2014). The growth condition of *B. distachyon* is simpler than rice. Furthermore, *B. distachyon* is more closely related in evolution to wheat than sorghum and rice (International *Brachypodium* Initiative, 2010), but unlike wheat with a complicated genome structure and large genome size (16,000 Mbp). *B. distachyon* has become an *Arabidopsis* rival particularly for crop plants. In this study, we isolated and characterized two *UBC13* genes in *B. distachyon*. The results of this study indicate that Ubc13 is highly conserved.

## Materials and Methods

### Plant Materials and Yeast Cell Culture

*Brachypodium distachyon 21* (Bd21) was used in this study. Bd21 seeds were surface sterilized with 20% sodium hypochlorite for 10 min, rinsed with sterile water five times, incubated in water for 12 h at room temperature, and then transferred to a wet filter paper to germinate in darkness for 24 h (22–25°C). The seeds that germinated uniformly were selected and spread in plastic pots containing ½ MS (Murashige and Skoog), which was changed every 2 days, and grown in a greenhouse with a daily photo cycle of 16 h light/8 h dark (26°C/18°C) and 65–75% air humidity.

The yeast strains used in this study are listed in Supplementary Table S1. Yeast cells were cultured at 30°C in YPAD or in a synthetic dextrose (SD) medium (0.67% Bacto-yeast nitrogen base without amino acids, 2% glucose) supplemented with necessary nutrients as recommended (Amberg et al., 2005). The lithium acetate method of yeast transformation was carried out as described (Ito et al., 1983). The sources and preparation of the *ubc13Δ::HIS3* (Brusky et al., 2000) cassette was as described previously.

### Molecular Cloning of *B. distachyon* cDNAs and Plasmid Construction

To clone *B. distachyon UBC13* genes, total RNA was extracted from *B. distachyon* seedlings using a TRIzol reagent (Invitrogen), and the ThermoScript RT-PCR kit (Invitrogen) was used to synthesize first-strand cDNA. The *BdUBC13* ORFs were amplified by PCR from the above cDNA preparation using gene-specific primers (Supplementary Table S2). The yeast two-hybrid vectors pGAD424Bg and pGBT9Bg were derived from pGAD424 and pGBT9 (Bartel and Fields, 1995). Genes cloned into plasmid pCAMBIA1300 are behind a CaMV 35S promoter.

### Yeast Two-Hybrid Analysis

The yeast two-hybrid strain PJ69-4a (James et al., 1996) was co-transformed with different Gal4_AD_ and Gal4_BD_ vectors. The co-transformed colonies were selected on an SD-Leu-Trp plate. Protein interaction was determined on synthetic complete medium lacking Trp, Leu and His, and supplemented with 3-amino-1,2,4-triazole (3-AT, Sigma-Aldrich).

### Protein Expression, Purification, and GST Pull-down Assay

Full-length *BdUBC13A* and *BdUBC13B* were cloned in plasmid pET30a(+). Full-length *AtUEV1A*, *AtUEV1B*, *AtUEV1C*, and *AtUEV1D* were cloned in plasmid pGEX-6p-1. The His_6_ and GST fusion constructs were transformed into *E. coli* strain BL21 (DE3). The recombinant proteins were purified with Ni Sepharose and glutathione (Amersham Pharmacia) according to the manufacturer’s protocol.

For the pull-down assay, crude cell extracts were loaded on Glutathione Sepharose^TM^ 4B beads and then 10 μg of purified His_6_-BdUbc13A or His_6_-BdUbc13B was later added. After incubation and washing, the GST beads were boiled with SDS-PAGE loading buffer for 10 min before western blotting analysis.

### Ub Conjugation Reaction

*In vitro* Ub conjugation reactions were performed by using the purified Ubc13A/B and GST-AtUev1A/C proteins as described above. Ub thioester conjugation initiation reagents were purchased from Boston Biochem. The 20 μl reaction mixture contained 225 nM E1 enzyme, 200 μM Ub, 1 mM MgATP, 1 mM Ubc13, and 1 mM Uev1 in the supplied reaction buffer. The K63R mutant Ub proteins were purchased from Boston Biochem (UM-K63R). The conjugation reactions were performed at 37°C for 2 h. Samples were subjected to SDS-PAGE (12%), and Ub and poly-Ub were detected by western blotting using mouse monoclonal anti-ubiquitin (P4D1; Cell Signaling).

### Yeast Survival Assay

Yeast strain HK578-10D and its isogenic *ubc13Δ* were transformed with pGAD-Ubc13A or pGAD-Ubc13B. Transformants were selected on SD-Leu plates. The gradient plate and serial dilution assays were performed as described previously (Xu et al., 2014). A gradient plate was made by pouring molten YPD agar with indicated MMS concentration into a tilted square Petri dish. After solidification, the Petri dish was returned flat and equal volume of YPD agar without MMS was poured to form the top layer. Cells from an overnight culture were mixed with molten YPD agar and immediately imprinted onto freshly made gradient plates via a microscope slide.

### Spontaneous Mutagenesis Assay

Yeast strain DBY747 derivative WXY849 bears a *trp1-289* amber mutation that can be reverted to Trp^+^ by several different mutation events (Xiao and Samson, 1993). DBY747 cells with the *ubc13Δ* mutation were transformed with pGAD-Ubc13A or pGAD-Ubc13B. The transformants were selected on SD-Leu plates. Each set of experiments contained five independent cultures of each strain. Overnight yeast cultures were counted using a hemocytometer and five replicate cultures of 10 ml of SD-Leu medium for each strain with a start concentration of 20 cells/ml were incubated at 30°C until the cell number reached 2 × 10^7^ cells/ml. Cells were collected by centrifugation at 4000 rpm, washed and plated onto YPAD in triplicate to score the total survivors and onto SD-Trp plates to score Trp^+^ revertants. Spontaneous mutation rates (number of revertants per cell per generation) were calculated as previously described (Williamson et al., 1985).

### Subcellular Localization of Ubc13s

*GFP-UBC13A* and *GFP-UBC13B* fusion genes were constructed by cloning *UBC13A* and *UBC13B* ORFs into the binary vector pCAMBIA1300-GFP downstream of GFP at the *Sac*I and *Kpn*I sites. The resulting constructs were introduced into *Agrobacterium tumefaciens* PMP90 then the PMP90 was co-infiltrated with *A. tumefaciens* P19 into *Nicotiana benthamiana* leaves (Waadt and Kudla, 2008). GFP and DAPI fluorescence were observed 2 days after transformation using a Zeiss 5 live confocal microscope.

### *B. distachyon* Protein Extraction and Western Blot Analysis

Different development phase samples were homogenized in liquid nitrogen. Total protein was extracted using a buffer containing 50 mM Tris-HCl pH 8.0, 0.3 M NaCl, 2 mM EDTA, 10% Glycerol, 0.1% Triton X-100, 10 mM PMSF, 3 mM DDT, and 1x protease inhibitor cocktail (Sigma-Aldrich). The extract was centrifuged at 16,000 *g* for 10 min at 4°C. The supernatant was then transferred to a new tube and boiled with SDS-PAGE loading buffer for 5 min before western blotting analysis. The BdUbc13 protein in each sample was detected by anti-hUbc13 monoclonal antibody 4E11 (Andersen et al., 2005).

## Results

### Identification and Sequence Analysis of the *BdUbc13* Genes

**FIGURE 1:**
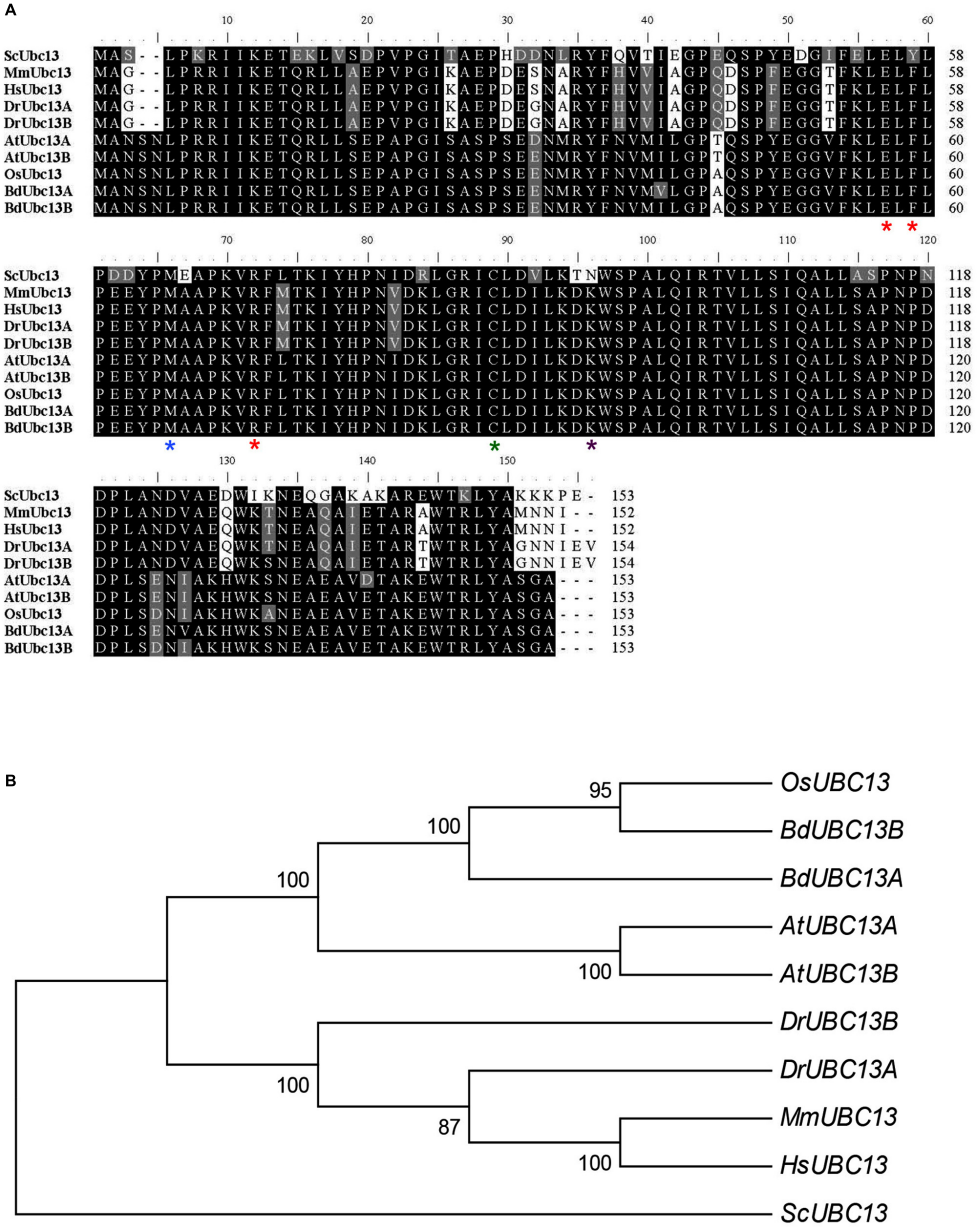
**Analysis of Ubc13 from different organisms. (A)** Amino acid sequence alignment of BdUbc13s and Ubc13s from other organisms. The sequences were aligned and edited using the BioEdit 7.0.9 program. Critical residues for Ubc13 functions are indicated with asterisks underneath the residue. Sc, *S. cerevisiae* (NP_010377); Mm, *Mus musculus* (NP_542127); Hs, *Homo sapiens* (NP_003339); Dr, *Danio rerio* (DrMUbc13a = NP_998651, DrUUbc13b = NP_956636); At, *A. thaliana* (AtUbc13A = NP_ 565289; AtUbc13B = NP_564011.1); Os, *Oryza sativa* Japonica Group (NP_001043834); Bd, *Brachypodium distachyon* (BdUbc13A = XP_003567909.1, BdUbc13B = XP_003569545.1). **(B)** Phylogenetic analyses of *UBC13* family coding DNA sequences (CDSs) from different organisms. The phylogenetic tree clustering was conducted with the neighbour joining method by using MEGA6.0.

To identify *B. distachyon UBC13* genes, two highly conserved *Arabidopsis UBC13* genes were used to blast the *B. distachyon* genomic database. Two genes, *BRADI2G16770* and *BRADI2G46290*, were obtained and named *BdUBC13A* and *BdUBC13B*, respectively. The two deduced BdUbc13 proteins contain only three amino-acid variations. In comparison with Ubc13s from other organisms, the active site Cys87 (green asterisk) for the Ub thioester formation (McKenna et al., 2001), Met64 (blue asterisk) for the physical interaction with RING finger E3 ligases (Wooff et al., 2004), and Glu55, Phe57, and Arg70 (red asterisks) for the physical interaction with Mms2 (Pastushok et al., 2005) are all conserved (**Figure 1A**). To further investigate the evolution of *B. distachyon UBC13*, the nucleotide sequences of *BdUBC13A* and *BdUBC13B* were compared with *UBC13* coding sequences from other organisms. There is 87% nucleotide sequence identity between *BdUBC13A* and *BdUBC13B*. The phylogenetic tree shows that *BdUBC13*s have evolved from a common plant ancestor and are closely related to *OsUBC13* (**Figure 1B**).

### Physical Interaction of BdUbc13s with Uve1s

**FIGURE 2:**
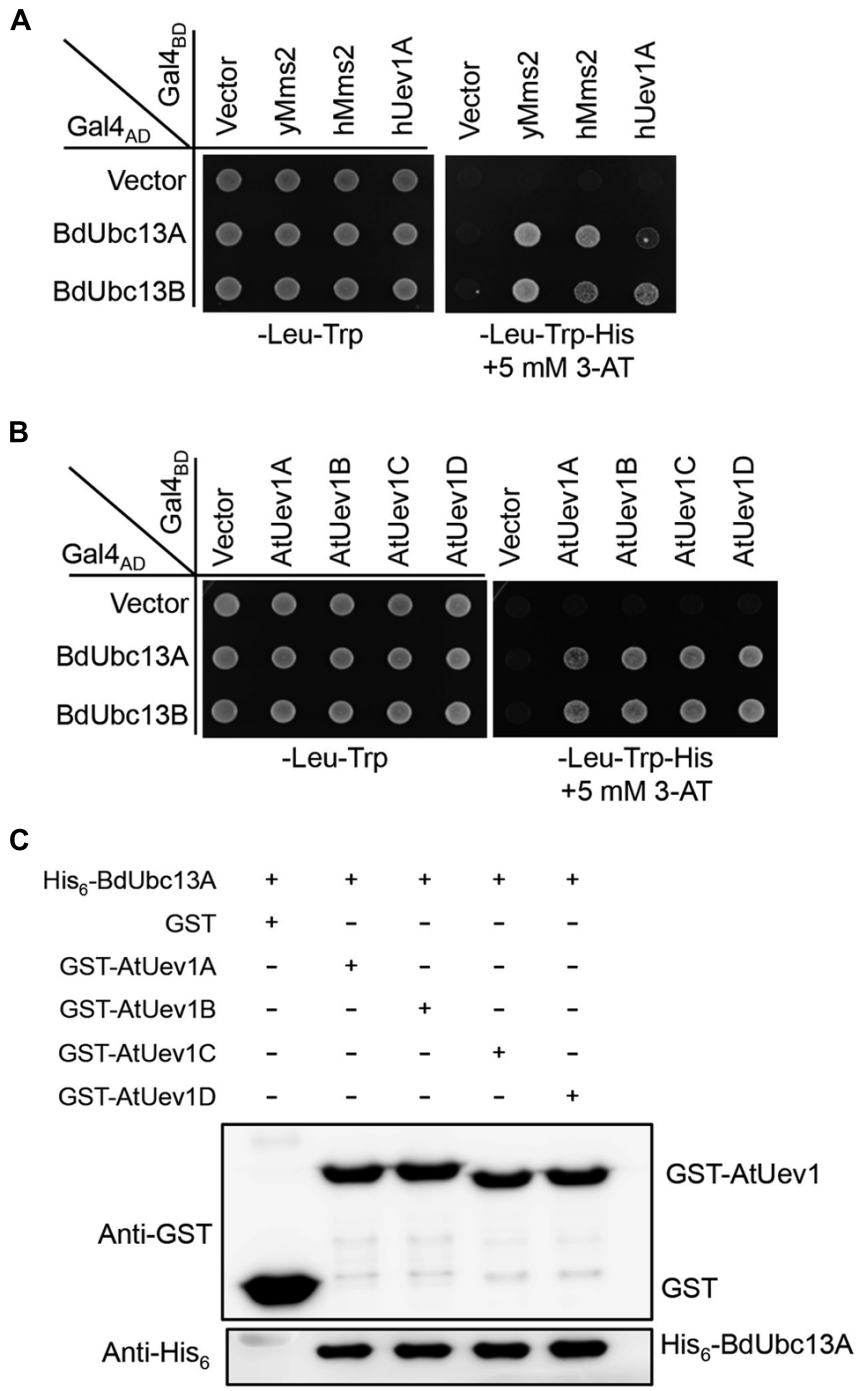
**BdUbc13s physically interact with Uev1s from other species. (A,B)** Physical interaction between BdUbc13s and Uevs in a yeast two-hybrid assay. PJ69-4a cells were transformed with BdUbc13 and Uev1 genes and the transformants carrying one Gal4_AD_ (pGAD424) and one Gal4_BD_ (pGBT9) plasmid were then selected, replicated onto various plates as indicated and incubated for 3 days before being photographed. The result is representative of at least five independent transformants from each treatment. **(C)** Protein interactions between BdUbc13A and *Arabidopsis* Uev1s by an affinity pull-down assay. BL21 (DE3) cells were transformed with pGEX-AtUev1s, and then the target gene expression was induced by adding 0.2 mM IPTG. Crude cell extracts were loaded on Glutathione Sepharose^TM^ 4B beads and 10 μg of purified His_6_-BdUbc13A was later added. After incubation and washing, the GST beads were boiled with the SDS-PAGE loading buffer for 10 min before western blotting analysis.

In budding yeast, Ubc13 and Mms2 form a heterodimer (Brusky et al., 2000) that synthesizes Lys63-linked polyubiquitin chains, which are required for error-free DDT (Broomfield et al., 2001). In human (Hofmann and Pickart, 1999), *Arabidopsis* (Wen et al., 2008) and Zebrafish (Wen et al., 2012), Ubc13s also interacts with Uev1s. A yeast two-hybrid assay (Fields and Song, 1989) was used to determine whether BdUbc13s interact with Uev1s. As seen in **Figure 2A**, the two BdUbc13s interact with yeast Uev1 (yMms2) and two human Uev1s (hMms2 and hUev1) in the presence of 5 mM 3-AT. They also have strong interaction with all four *Arabidopisis* Uev1s (**Figure 2B**). To further confirm the physical interaction between BdUbc13s and Uev1s *in vitro*, a GST-affinity pull-down assay was performed. As shown in **Figure 2C** and Supplementary Figure S1A, recombinant GST-AtUev1s fusion proteins can pull down purified recombinant His_6_-BdUbc13 fusion proteins, but the GST alone cannot. Based on the above observations, we conclude that BdUbc13s can form stable heterodimers with Uev1s *in vivo* and *in vitro*.

### BdUbc13s Mediates K63-Linked Polyubiquitination with *Arabidopsis* Uevs *In Vitro*

**FIGURE 3:**
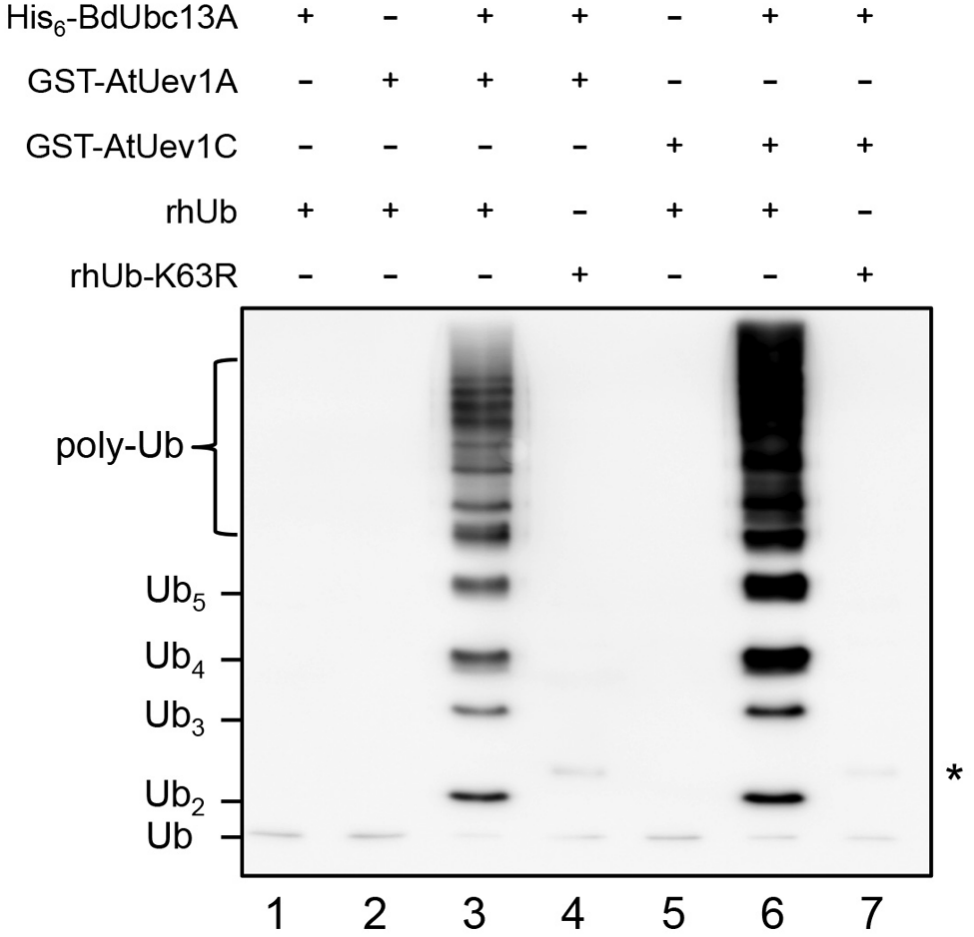
***In vitro* ubiquitin conjugation assays of BdUbc13A using purified proteins**. After ubiquitination reactions as described, samples were subjected to SDS-PAGE and western blotting using an anti-Ub antibody to monitor poly-Ub chain formation. rhUb, recombinant human ubiquitin. ^∗^ indicates the non-specific bands in lanes 4 and 7.

It is well known that Ubc13 and Mms2 (Uev) play a key role in the DDT pathway by polyubiquitinating PCNA with K63-linked chains (Hoege et al., 2002). Ubc13 and Uev1 together can catalyze K63-linked free Ub chains (Hofmann and Pickart, 1999). However, hUbc13-K92 serves as the site of Ub conjugation and a hUbc13-K92A mutation does not affect the free chain formation (McKenna et al., 2001). To avoid Ub conjugation to Ubc13 itself, we made the corresponding BdUbc13-K94A mutation (**Figure 1A**, purple asterisk) and found that BdUbc13A together with AtUev1A or AtUev1C can indeed generate free Ub chains while either BdUbc13 or AtUev1 alone cannot (**Figure 3**). More importantly, if Ub-K63R was used in the same reaction, no poly-Ub chains were observed, confirming that they are indeed K63-linked poly-Ub chains. The same result was also obtained for BdUbc13B together with AtUev1A or AtUev1C (Supplementary Figure S1B). Hence, *B. distachyon* Ubc13s and AtUev1s catalyze Lys63-linked Ub chains *in vitro*.

### Functional Complement of Yeast *ubc13* Mutant by *BdUbc13s*

**FIGURE 4:**
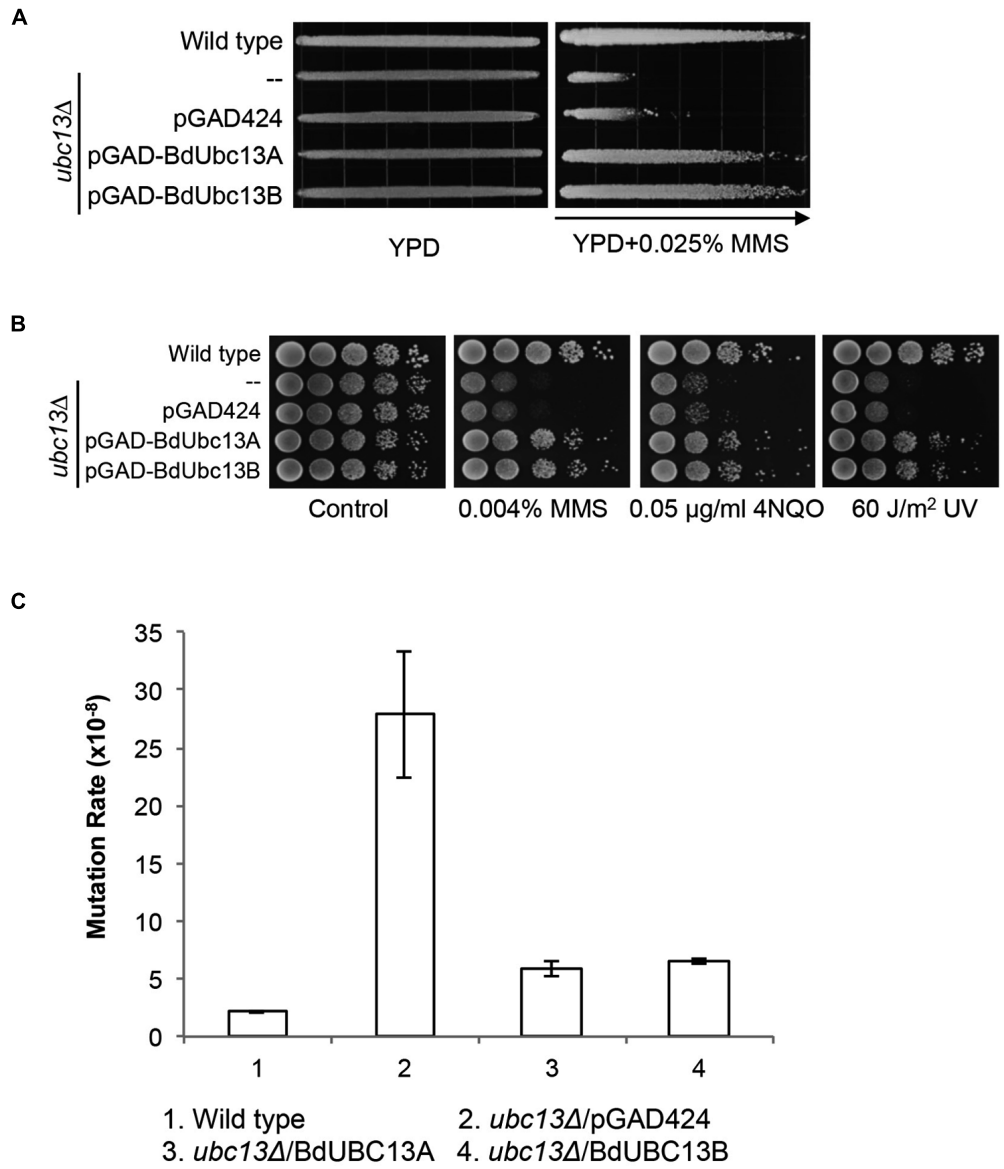
**Functional complementation of the yeast *ubc13* null mutant by *BdUBC13s.* (A)** Complementation of the *ubc13* single mutant by *BdUBC13A* and *BdUBC13B*. Yeast strain HK578-10D (wild type) and the *ubc13*Δ transformants (WXY904 transformed with pGAD-BdUbc13A or pGAD-BdUbc13B) were grown overnight and then printed onto the gradient plate. The YPD control (left) and YPD+0.025% MMS gradient (right) plates were incubated at 30°C for 3 days. Arrow points to gradually increasing MMS concentrations. **(B)** Functional complementation of the *ubc13* null mutant by *BdUBC13A* and *BdUBC13B* from representative DNA-damaging agents by a serial dilution assay. Yeast strains as indicated were grown overnight in SD selective media, diluted and treated with the DNA-damaging agents. Yeast strains used: HK578-10D (wild type) and WXY904 (*ubc13Δ*). **(C)** Spontaneous mutation rates of *S. cerevisiae ubc13* mutants. All strains are isogenic derivatives of DBY747. The results are the average of three independent experiments with standard deviations. All the genes were cloned in pGAD424.

Yeast Ubc13 and Mms2 function in the error-free DDT pathway (Broomfield et al., 1998; Brusky et al., 2000). To determine whether the BdUbc13s also play critical roles in error-free DDT, we took advantage of the *UBC13* gene conservation within eukaryotes and performed a gradient plate assay (Xiao et al., 1996) and spontaneous mutagenesis assay (Xiao and Samson, 1993). As shown in **Figure 4A**, the expression of *BdUBC13A* or *BdUBC13B* genes can protect the *ubc13* null mutant from killing by MMS, while the pGAD424 vector cannot.

The DDT pathway differs from other DNA repair pathways in that its defect induces hypersensitivity to a wide range of DNA-damaging agents that cause replication blocks. To further confirm that *BdUBC13s* function in the yeast DDT pathway, we performed a serial dilution assay in the presence of several representative DNA-damaging agents including MMS (an alkylating agent), UV irradiation, and 4-nitroquinoline 1-oxide (4NQO, causes bulky adducts). As shown in **Figure 4B**, both *BdUbc13A* and *BdUbc13B* can rescue the *ubc13 null* mutant phenotypes while the vector transformant has no effect in the phenotypic rescue.

One of the hallmarks of error-free DDT is its ability to limit spontaneous and DNA-damage-induced mutagenesis (Xu et al., 2015). Under our experimental conditions, the *ubc13* mutant displayed a 13-fold increase in the spontaneous mutation rate. The expression of *BdUBC13A* or *BdUBC13B* reduced the spontaneous mutation rate of the *ubc13* null mutant to nearly the wild type level (**Figure 4C**), and the slight difference with the wild-type rate could be accounted for by plasmid loss in the transformants. Taken together, our results indicate that BdUbc13 can replace the function of yUbc13 in the yeast error-free DDT pathway.

### BdUbc13 Subcellular Distribution

**FIGURE 5:**
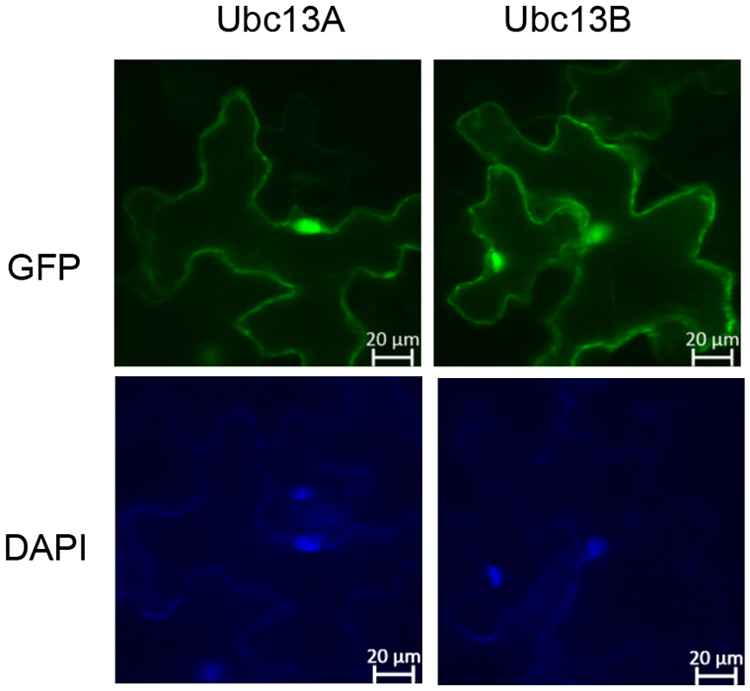
**Subcellular localization of GFP-BdUbc13s. (Top)** Transient expression of GFP-tagged BdUbc13s in tobacco epidermal cells visualized by epifluorescence microscopy. **(Bottom)** DAPI staining to visualize nuclei.

In order to assess possible BdUbc13 functions in its native host, we examined the subcellular distribution of BdUbc13s in tobacco. GFP-BdUbc13A and GFP-BdUbc13B fusion constructs were created, transiently transfected to tobacco (*N. benthamiana*) leaves by *A. tumefaciens* and their subcellular localization was monitored by fluorescence microscopy. As shown in **Figure 5**, both GFP-BdUbc13A and GFP-BdUbc13B were found in the nucleus and cytoplasm, but appear to be enriched in the nucleus. In addition, the fluorescent signals were also readily detected in the cytoplasmic membrane. Since, the DDT function occurs exclusively in the nucleus, the above observations suggest that BdUbc13A and BdUbc13B are also involved in other cellular pathways in addition to DDT.

### *BdUBC13* Expression During Development and in Response to Abiotic Stresses

**FIGURE 6:**
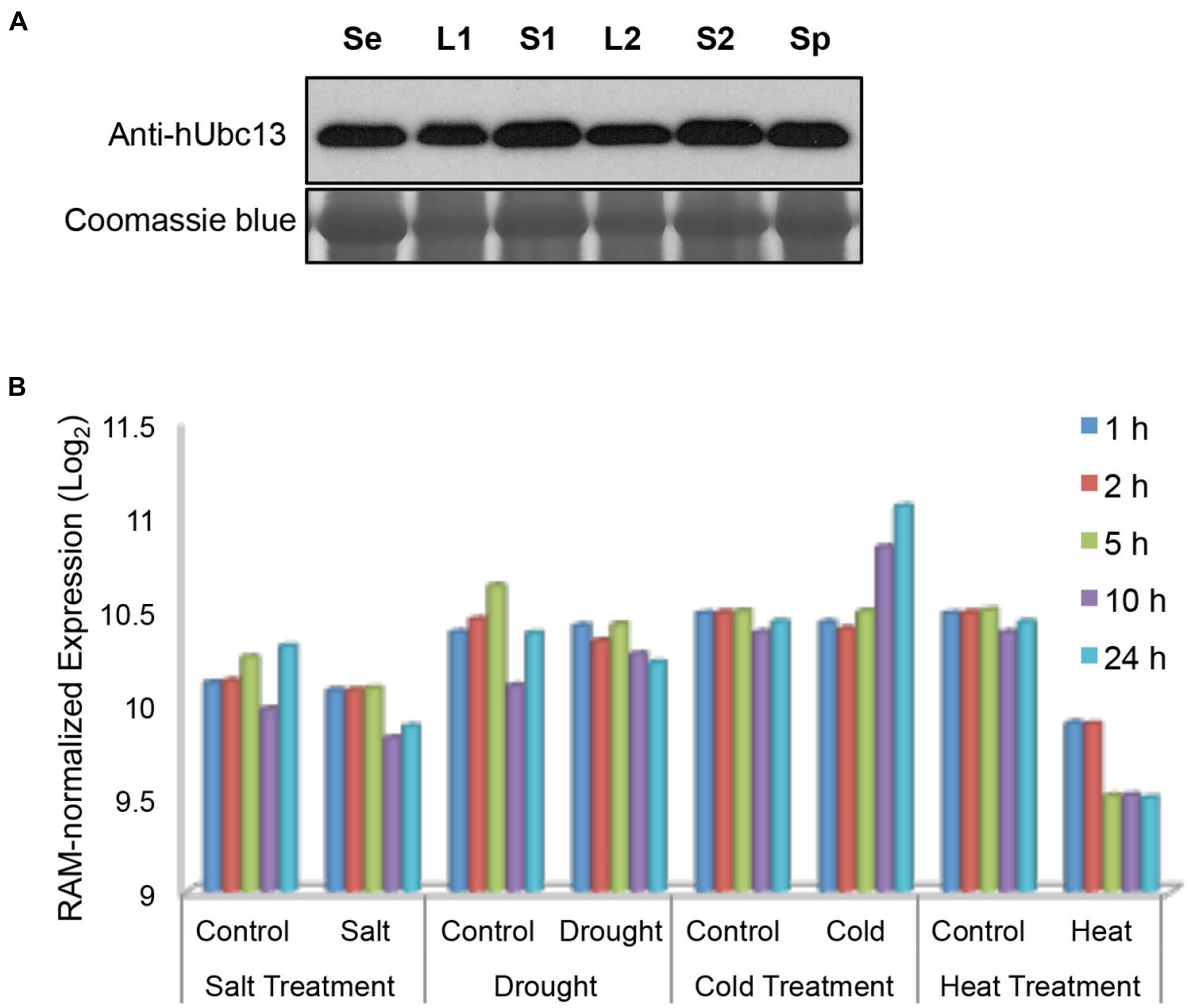
**Quantitative analysis of *BdUBC13* expression. (A)** BdUbc13 protein levels in different tissues during *B. distachyon* development. Samples were taken from different developmental stages and tissues as indicated. Se: 7-days seedling; L1 and S1: leaves and stems from transition phase; L2 and S2: the top leaves and stems from advanced phase; Sp: spikes. Coomassie staining of the SDS-PAGE gel serves as a loading control. **(B)** Relative expression of *BdUBC13A* under different abiotic stresses. Original data are extracted from the *Brachypodium* genome database (http://jbrowse.brachypodium.org).

It was reported that the *Arabidopsis UBC13* gene has a relatively uniform expression in different tissues or during biotic or abiotic stresses (Wen et al., 2006), and *OsUBC13* expression remains remarkably constitutive during development (Zang et al., 2012). To investigate *BdUBC13* expression pattern during development, total protein was extracted from 7-days seedling as well as different tissues and subjected to western blot analysis using a monoclonal antibody raised against human Ubc13 (Andersen et al., 2005). As seen in **Figure 6A**, total BdUbc13 levels (Ubc13A + Ubc13B) do not appear to fluctuate dramatically during development and in different tissues.

We also analyzed relative *BdUBC13* expression in response to abiotic stress from an available database (Priest et al., 2014). It is of great interest to note that while salt and drought stresses do not significantly alter *BdUBC13A* expression, cold stress induces *BdUBC13A* over time and heat treatment dramatically reduces *BdUBC13A* expression (**Figure 6B**), suggesting the involvement of *BdUBC13A* in response to environmental temperature stresses. Unfortunately there was no data on *BdUBC13B* in the same database.

## Discussion

In this study, we isolated and characterized two *UBC13* genes from *B. distachyon*. Sequence analyses indicate that the *B. distachyon* genome contains two highly conserved *UBC13*s. The *in vivo* results in this study revealed that both *BdUBC13A* and *BdUBC13B* could rescue the yeast *ubc13* null mutant phenotypes to tolerate DNA-damaging agents and reduce spontaneous mutagenesis, characteristic of error-free DDT. Since physical interactions of Ubc13 with yeast Mms2 and Rad5 are absolutely required for its error-free DDT activity in budding yeast (Xu et al., 2015), the above results indicate that BdUbc13 must be able to bind yeast Mms2 and Rad5 to form a functional E2–E3 complex. In addition, BdUbc13s can form heterodimers with Uevs from other species including *Arabidopsis*, suggesting that K63-linked polyubiquitination is a highly conserved process within eukaryotes.

The two BdUbc13 proteins are highly conserved with only three amino-acid variations, all of which are conserved amino acid substitutions falling in the variable residues among different species. Hence, it is unlikely that the two copies of *BdUBC13* genes function differently. Since phylogenic analysis indicates that the two *BdUBC13* genes were derived from gene duplication and one of them was later lost in rice, one would assume that the two *BdUBC13* genes are functionally redundant, although this speculation needs to be critically examined when individual mutant lines are made available.

In budding yeast, the only defined function of Ubc13-Mms2 is to polyubiquitinate PCNA after its monoubiquitination by Rad6–Rad18 (Hoege et al., 2002); this polyubiquitination signals for error-free lesion bypass via template switch (Ball et al., 2009). In mammalian cells, Ubc13 works with different Uev partners Mms2 and Uev1A to be involved in DNA-damage response and NF-κB signaling, respectively (Andersen et al., 2005). It is hypothesized that in multicellular organisms, Uevs may serve as a regulatory subunit and dictate which target protein(s) will be polyubiquitinated by the Ubc13-Uev complex. This hypothesis predicts that Ubc13 is an abundant protein and distributed to different subcellular locations. Indeed, this and previous studies revealed that *UBC13* is constitutively expressed at high levels in all plant tissues and developmental stages. In addition, this is the first report to our knowledge showing that Ubc13 is localized to the nucleus and cytoplasm, as well as the periplasmic membrane, indicating that plant Ubc13 is a multi-functional protein. In *Arabidopsis*, all four Uev1s are capable of binding Ubc13, but only Uev1D is known to be involved in DNA-damage response (Wen et al., 2008), while the functions of other three Uev1s remain to be revealed. Since the *B. distachyon* genome also contains several Uev1 homologs (data not shown), it is conceivable that BdUbc13 may form heterodimers with different Uev1s to be involved in different signaling pathways. These pathways may include, but are not limited to, apical dominance (Yin et al., 2007), iron metabolism (Li and Schmidt, 2010), immunity (Mural et al., 2013), and auxin signaling (Leitner et al., 2012; Wen et al., 2014).

The K63-linked polyubiquitination process is primarily reported to be involved in stress responses, such as DNA damage, oxidative stress, and immunity. Budding yeast *UBC13* is a DNA-damage-inducible gene (Brusky et al., 2000). The transcriptional regulation of *UBC13* in mammalian cells has not been fully explored, although a recent report showed that *UBC13* is suppressed by STAT3 to regulate its NF-κB activity (Zhang et al., 2014). The expression of *UBC13* appears to be constitutive in *Arabidopsis* (Wen et al., 2006), rice (Zang et al., 2012), and *B. distachyon* at different developmental stages and in different tissues. Since both *Arabidopsis* and *B. distachyon* genomes contain two *UBC13* genes and their promoter sequences are rather different (Wen et al., 2006; data not shown), one cannot rule out a possibility that the two genes have distinct expression patterns. In contrast, the expression of plant *UEV1* genes fluctuates dramatically under the same experimental conditions (Wen et al., 2008), suggesting that the Uev1 subunit controls the Ubc13-Uev E2 enzyme activity by regulating the Uev1 tissue-specific expression and subcellular localization. Surprisingly, *BdUBC13A* appears to be induced by cold and suppressed by heat treatment, suggesting its involvement in the regulation of temperature response. Unfortunately, due to the limited data availability in the *B. distachyon* database, we were unable to readily assess *BdUBC13B* expression in response to abiotic stresses. Nevertheless, this study in combination with *B. distachyon* serving as an ideal model organism for monocots and temperate cereals will promote research in this unconventional ubiquitination field and its applications to agriculture and food safety.

## Author Contributions

Experimental design: HG, RW, WX; Experiments: HG, RW, ZL; manuscript preparation: HG, WX; Supervision, funding, and reagents: WX, RD.

## Conflict of Interest Statement

The authors declare that the research was conducted in the absence of any commercial or financial relationships that could be construed as a potential conflict of interest. The reviewer Wolfgang Schmidt and handling Editor Keqiang Wu declared their collaboration, and the handling Editor states that, nevertheless, the process met the standards of a fair and objective review.
