# Supplementary material for: Molecular Cloning and Functional Characterization of Two Brachypodium distachyon UBC13 Genes Whose Products Promote K63-Linked Polyubiquitination
Source: Front Plant Sci. 2016 Jan 7;6:1222. doi: 10.3389/fpls.2015.01222 (PMC4703986; doi:10.3389/fpls.2015.01222)
Supplement: Supplementary file 1 [file Image_1.PDF]

Supplementary Figure and Tables

Table S1. ***Saccharomyces cerevisiae*** **strains**

Strain	Genotype	Source
PJ69-4A	*MATa trp1-901 leu2-3,112 ura3-52 his3-200 gal4Δ gal80Δ Met2::GAL7-lacZ LYS2::GAL1-HIS3 GAL2-ADE2*	P. James
HK578-10D	*MATa ade2-1 can1-100 his3-11,15 leu2-3,112 trp1-1 ura3-1*	H. Klein
WXY904	HK578-10D with *ubc13Δ::HIS3*	This study
DBY747	MATa *his3-1 leu2-3, 112 trp1-289 ura3-52*	D. Botstein
WXY849	DBY747 with *ubc13Δ::HIS3*	This study

Table S2. Primers used for plasmid constructions

<table>
<tr><th>plasmid</th><th>Forward Primers</th><th>Reverse Primers</th></tr>
<tr><td>pGAD-Ubc13A</td><td rowspan="4">CCGGAATTCATGGCCAACAGCAACCTCCCG</td><td rowspan="2">ACGCGTCGACTCAAGCACCACTGGCATACAG</td></tr>
<tr><td>pET30a-Ubc13A</td></tr>
<tr><td>pGAD-Ubc13B</td><td rowspan="2">ACGCGTCGACTCATGCGCCACTCGCATACAG</td></tr>
<tr><td>pET30a-Ubc13B</td></tr>
<tr><td>pCAMBIA1300-Ubc13A-GFP</td><td rowspan="2">ACCGAGCTCATGGCCAACAGCAACCTCCCG</td><td>CGGGGTACCTCAAGCACCACTGGCATACAG</td></tr>
<tr><td>pCAMBIA1300-Ubc13B-GFP</td><td>CGGGGTACCTCATGCGCCACTCGCATACAG</td></tr>
</table>

Figure S1. Physical interaction and *in vitro* ubiquitination promoted by BdUbc13B. (A) Protein interactions between BdUbc13B and Arabidopsis Uev1s by an affinity pull-down assay. BL21 (DE3) cells were transformed with pGEX-AtUev1As and then the target gene expression was induced by adding 0.2 mM IPTG. Crude cell extracts were loaded on Glutathione Sepharose^{TM} 4B beads and 10 μg of purified His_6-BdUbc13B was later added. After incubation and washing, the GST beads were boiled with SDS-PAGE loading buffer for 10 min before western blotting analysis. (**B**) *In vitro* ubiquitin conjugation assays of BdUbc13B using purified proteins. After ubiquitination reactions as described, samples were subjected to SDS-PAGE and a western blot using an anti-Ub antibody was performed to monitor poly-Ub chain formation. * indicates the nonspecific band in lanes 4 and 7.

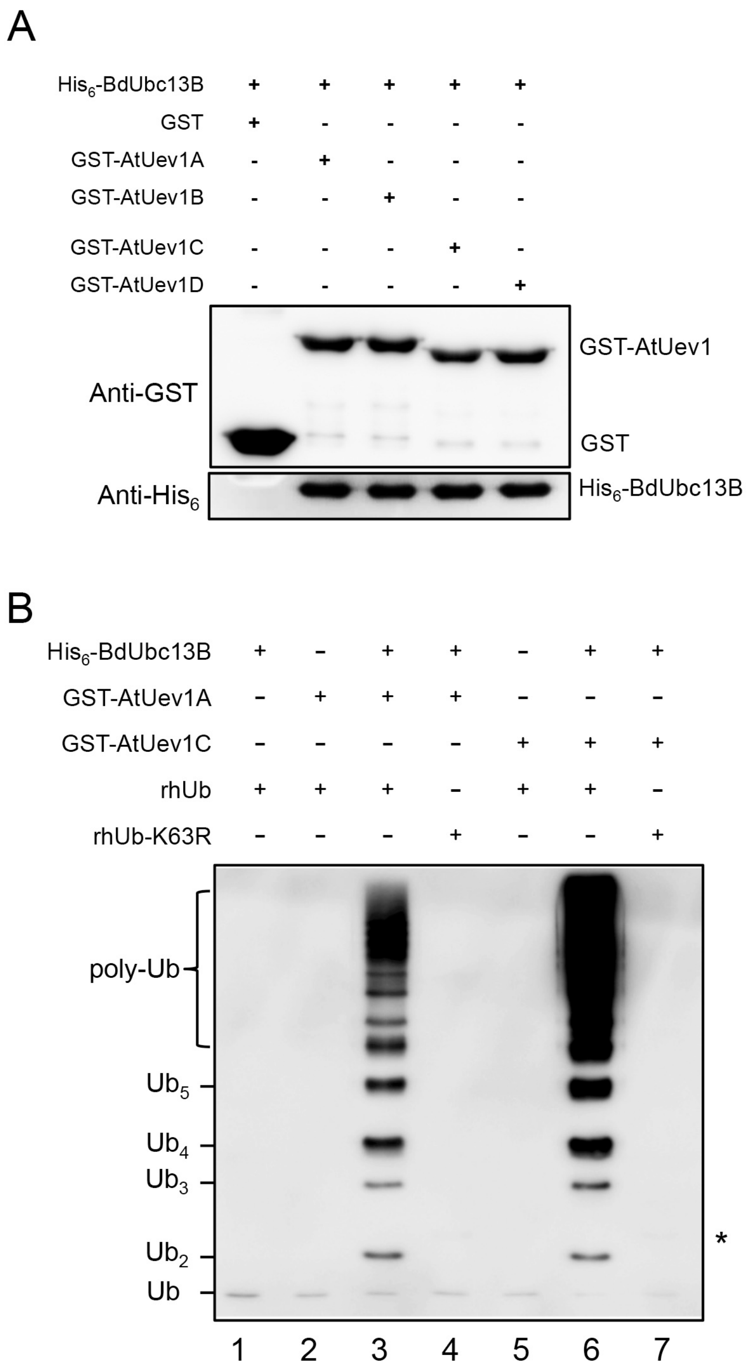
A
His6-BdUbc13B
GST
GST-AtUev1A
GST-AtUev1B
GST-AtUev1C
GST-AtUev1D
Anti-GST
GST-AtUev1
GST
Anti-His6
His6-BdUbc13B
B
His6-BdUbc13B
GST-AtUev1A
GST-AtUev1C
rhUb
rhUb-K63R
poly-Ub
Ub5
Ub4
Ub3
Ub2
Ub
*
1
2
3
4
5
6
7

Figure S1
